# Poly[[2-(3-pyridinio)-1*H*,3*H* ^+^-benzimidazolium] [μ_4_-oxido-di-μ_3_-oxido-tetra-μ_2_-oxido-hexa­oxidotetra­molybdenum(VI)]]

**DOI:** 10.1107/S160053680901294X

**Published:** 2009-04-10

**Authors:** Li-Juan Chen, Shen Lin, Xiao-Yuan Wu, Xiao-Hua Chen

**Affiliations:** a College of Chemistry and Materials Science, Fujian Normal University, Fuzhou, Fujian 350007, People’s Republic of China; b State Key Laboratory of Structural Chemistry, Fujian Institute of Research on the Structure of Matter, The Chinese Academy of Science, Fuzhou, Fujian 350002, People’s Republic of China

## Abstract

The reaction of MoO_3_ with 2-(3-pyrid­yl)benzoimidazole and water in the presence of MnSO_4_·5H_2_O at 453 K under hydro­thermal conditions afforded the title compound, {(C_12_H_11_N_2_)[Mo_4_O_13_]}_*n*_, in which infinite molybdenum oxide anionic chains are charge-balanced by diprotonated 2-(3-pyrid­yl)benzoimidazole (H_2_3-PBIM^2+^) cations. Eight [MoO_6_] octa­hedra are edge-shared, forming compact octa­molybdate subunits which are connected through pairs of Mo—O—Mo bridges into extended one-dimensional arrays propagating along the *a-*axis direction. The asymmetric unit of the metal oxide chain contains one half of the octa­molybdate unit, denoted [Mo_4_O_13_], the other half being generated by an inversion center. These molybdenum oxide chains are further connected through the 2-(3-pyridinio)benzoimidazolium cations into a three-dimensional network *via* N—H⋯O hydrogen bonds. In addition, neighbouring diprotonated cations are arranged in a head-to-tail fashion with a plane-to-plane separation of 3.63 (10) Å, indicating the existence of weak aromatic π–π stacking inter­actions.

## Related literature

For the properties, applications and reactivity of inorganic-organic hybrid materials, see: Pope (1983 ▶); Pope & Müller (1991 ▶); Kong *et al.* (2004 ▶). For chain, sheet and framework structural types, see: Hagrman *et al.* (1999 ▶); Lu *et al.* (2002 ▶). For related structures, see: Chakrabarti & Natarajan (2002 ▶); Janiak (2000 ▶); Modec *et al.* (2004 ▶); Xiao *et al.* (2005 ▶).
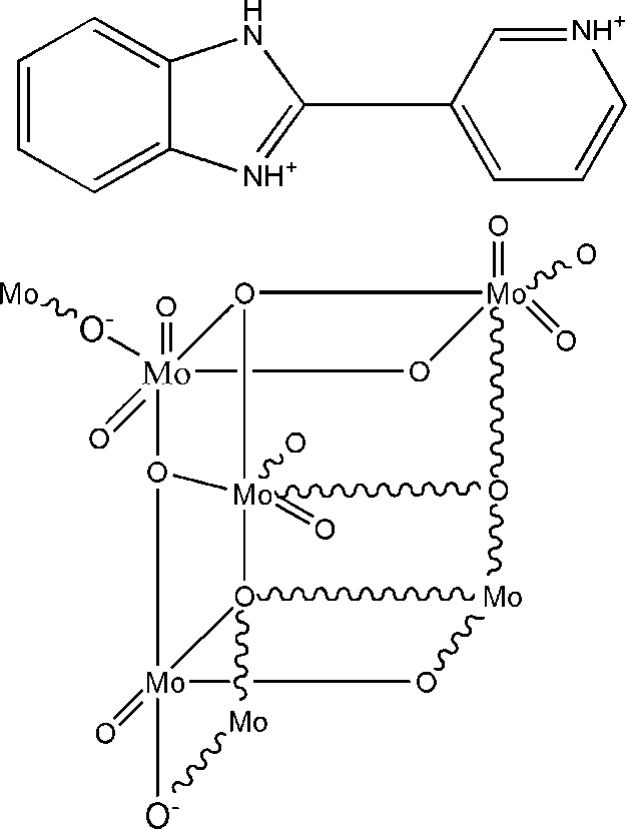


## Experimental

### 

#### Crystal data

(C_12_H_11_N_2_)[Mo_4_O_13_]
*M* *_r_* = 789.00
Triclinic, 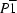

*a* = 7.947 (3) Å
*b* = 11.503 (5) Å
*c* = 11.630 (5) Å
α = 70.038 (14)°
β = 76.856 (17)°
γ = 75.947 (17)°
*V* = 957.2 (7) Å^3^
*Z* = 2
Mo *K*α radiation
μ = 2.64 mm^−1^
*T* = 293 K
0.10 × 0.05 × 0.02 mm

#### Data collection

Rigaku Mercury CCD diffractometer
Absorption correction: multi-scan (*CrystalClear*; Rigaku, 2002 ▶) *T*_min_ = 0.763, *T*_max_ = 0.949
6127 measured reflections
3358 independent reflections
2594 reflections with *I* > 2σ(*I*)
*R*_int_ = 0.047

#### Refinement

*R*[*F* ^2^ > 2σ(*F* ^2^)] = 0.043
*wR*(*F* ^2^) = 0.100
*S* = 1.01
3358 reflections
289 parameters
6 restraints
H-atom parameters constrained
Δρ_max_ = 0.98 e Å^−3^
Δρ_min_ = −1.14 e Å^−3^

### 

Data collection: *CrystalClear* (Rigaku, 2002 ▶); cell refinement: *CrystalClear*; data reduction: *CrystalClear*; program(s) used to solve structure: *SHELXS97* (Sheldrick, 2008 ▶); program(s) used to refine structure: *SHELXL97* (Sheldrick, 2008 ▶); molecular graphics: *SHELXTL* (Sheldrick, 2008 ▶) and *DIAMOND* (Brandenburg, 1999 ▶)’; software used to prepare material for publication: *SHELXL97*.

## Supplementary Material

Crystal structure: contains datablocks global, I. DOI: 10.1107/S160053680901294X/sj2606sup1.cif

Structure factors: contains datablocks I. DOI: 10.1107/S160053680901294X/sj2606Isup2.hkl

Additional supplementary materials:  crystallographic information; 3D view; checkCIF report

## Figures and Tables

**Table 1:** Selected bond lengths (Å)

Mo1—O2	1.690 (5)
Mo1—O11	1.776 (5)
Mo1—O9	1.875 (5)
Mo1—O10^i^	1.956 (5)
Mo1—O6	2.189 (5)
Mo1—O10	2.416 (5)
Mo2—O5	1.693 (5)
Mo2—O4	1.709 (5)
Mo2—O8	1.927 (5)
Mo2—O3	2.004 (5)
Mo2—O10	2.200 (5)
Mo2—O9	2.345 (5)
Mo3—O7	1.699 (5)
Mo3—O13	1.790 (5)
Mo3—O6^i^	1.880 (5)
Mo3—O3	1.922 (5)
Mo3—O11	2.229 (6)
Mo3—O10	2.242 (5)
Mo4—O1	1.682 (5)
Mo4—O12	1.719 (5)
Mo4—O8	1.965 (5)
Mo4—O13^ii^	2.012 (5)
Mo4—O6	2.160 (5)
Mo4—O9	2.266 (5)

Symmetry codes: (i) 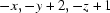; (ii) 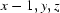.

**Table 2:** Hydrogen-bond geometry (Å, °)

*D*—H⋯*A*	*D*—H	H⋯*A*	*D*⋯*A*	*D*—H⋯*A*
N1—H1*A*⋯O3^iii^	0.86	1.78	2.639 (8)	176
N3—H3*A*⋯O8	0.86	1.78	2.614 (8)	164

Symmetry code: (iii) 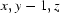.

**Fig. 1.**
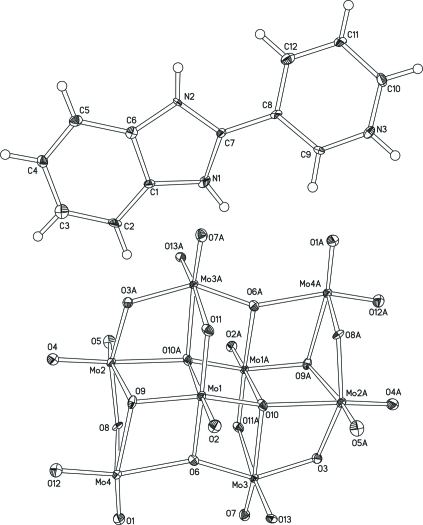
A molecular drawing of (I), showing 30% probability displacement ellipsoids.

**Fig. 2.**
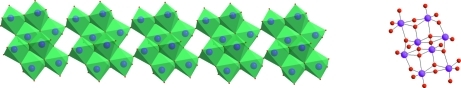
A polyhedral representation of the infinite chain in title compound and the γ-[Mo8O26]4-. All C, N and H atoms were omitted for clarity.

**Fig. 3.**
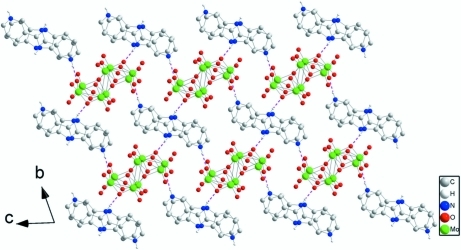
Packing diagram of title compound along a axis. Broken lines indicate hydrogen bonds. All H atoms, which do not participate in the bydrogen bonds, have been omitted for clarity.

## supplementary crystallographic information

### Comment

The exploration of metal oxide-based inorganic-organic hybrid materials is of contemporary interest in the fields of solid state chemistry, not only because of their fascinating properties and potential applications in many fields, such as catalysis, sorption, electrical conductivity, magnetism and optical materials (Pope & Müller, 1991, Pope, 1983). Owing to their versatile stoichiometry, different structure, and high reactivity (Kong, 2004), molybdenum polyoxoanions are good candidates to function as building blocks for inorganic-organic hybrid materials. Through exploiting the strategy of synergistic interaction between organic and inorganic components, many examples of molybdenum oxide-based solid materials with one-dimensional chain, two-dimensional sheet and three-dimensional framework structures have been successfully synthesized (Hagrman *et al.*, 1999, Lu *et al.*, 2002). The organic components often function as charge compensating cations or as a linking bridges, to extend the molybdenum oxide building units into multi-dimensional networks. We report here the synthesis and crystal structure of the title compound, in which the organic component acts as a charge compensating cation.

The structure of title compound consists of an infinite molybdenum oxide chain which is charge balanced by diprotonated H_2_3-PBIM^2+^ cations. As shown Fig. 1, every Mo atom is coordinated octahedrally by six O atoms. These can be divided into four groups according to their coordination environments: (i) Mo—O(*t*), 1.682 (5)–1.718 (5) Å; (ii) Mo—O(µ_2_-O), 1.776 (5)–2.229 (6) Å; (iii) Mo—O(µ_3_-O), 1.875 (5)- 2.345 (5) Å; (iv) Mo—O(µ_4_-O), 1.956 (5)–2.416 (5) Å (Table 1).

The asymmetric unit of the metal oxide chain contains one half of the octamolybdate unit, denoted as [Mo_4_O_13_], the other half is generated by the inversion center. Four asymmetric [MoO_6_] octahedra are edge- shared to form [Mo_4_O_13_]^2-^ unit. Two [Mo_4_O_13_]^2-^ units are stacked together by edge-sharing to give rise to γ-[Mo_8_O_26_]^4-^ octamolybdate clusters, which are linked together to form infinite one- dimensional chains propagating along the *a*-direction through sharing pairs of common vertices. Therefore, the molybdenum oxide chain may be regarded to be constructed from octamolybdate units joined at two oxo groups or from two groups of *cis*-edge-sharing tetranuclear units fused at two common corners. The octamolybdate chain in the title compound is structurally analogous to those found in [Me—NC_5_H_5_]_4_[Mo_8_O_26_] (Modec *et al.*, 2003), [H_2_enMe]_2_[Mo_8_O_26_] (Xiao *et al.*, 2005), and [NH_3_(CH_2_)_2_NH_3_]_2_[Mo_8_O_26_] (Chakrabarti & Natarajan, 2002).

In the solid state of the title compound, the one-dimensional molybdenum oxide chains are held together and extended to three-dimensional framework *via* strong N—H···O hydrogen bonding and weak aromatic π-π stacking interactions. As illustrated in Fig. 3, one of the imino groups and the pyridyl group in the H_2_3-PBIM^2+^ ligands participate in the intermolecular hydrogen bonding with two µ_2_-O atoms of the molybdenum oxide chains. The N···O separations are 2.639 (8) and 2.614 (8) Å with both H···O distances are 1.78 Å, falling into the normal range of the strong hydrogen bond interactions. The bond angles are 176.3 and 163.6 °, respectively. In addition, the neighbouring diprotonated H_2_3-PBIM^2+^ ligands along the *a*-direction are arranged in a head-to-tail fashion with a plane-to-plane separation of 3.63 (10) Å, indicating the existence of weak aromatic π-π stacking interactions (Janiak, 2000).

### Experimental

A mixture of MoO_3_, MnSO_4_.5H_2_O, 2-(3-pyridyl)benzoimidazole and H_2_O in the molar ratio 1.0:1.2:1.0:1835 was sealed in a 18 ml Teflon-lined Parr acid digestion bomb and heated for 3 days at 453 K and autogeneous pressure. After allowing the reaction mixture to cool down to room temperature, colorless needle-like crystals of title compound were collected, washed with water and air dried.

### Refinement

The positions of all hydrogen atoms were generated geometrically (C—H and N—H bonds fixed at 0.96 Å and 0.86 Å, respectively), assigned isotropic thermal parameters, and allowed to ride on their respective parent C or N atoms before the final cycle of least-squares refinement.

### Crystal data

**Table:**

(C_12_H_11_N_2_)[Mo_4_O_13_]	*Z* = 2
*M**_r_* = 789.00	*F*(000) = 752
Triclinic, *P*1	*D*_x_ = 2.738 Mg m^−^^3^
Hall symbol: -P 1	Mo *K*α radiation, λ = 0.71073 Å
*a* = 7.947 (3) Å	Cell parameters from 2049 reflections
*b* = 11.503 (5) Å	θ = 3.0–25.0°
*c* = 11.630 (5) Å	µ = 2.64 mm^−^^1^
α = 70.038 (14)°	*T* = 293 K
β = 76.856 (17)°	Prism, colorless
γ = 75.947 (17)°	0.10 × 0.05 × 0.02 mm
*V* = 957.2 (7) Å^3^	

### Data collection

**Table:**

Rigaku Mercury CCD diffractometer	3358 independent reflections
Radiation source: fine-focus sealed tube	2594 reflections with *I* > 2σ(*I*)
graphite	*R*_int_ = 0.047
Detector resolution: 14.6306 pixels mm^-1^	θ_max_ = 25.0°, θ_min_ = 3.0°
ω scans	*h* = −9→9
Absorption correction: multi-scan (*CrystalClear*; Rigaku, 2002)	*k* = −13→12
*T*_min_ = 0.763, *T*_max_ = 0.949	*l* = −13→13
6127 measured reflections	

### Refinement

**Table:**

Refinement on *F*^2^	Primary atom site location: structure-invariant direct methods
Least-squares matrix: full	Secondary atom site location: difference Fourier map
*R*[*F*^2^ > 2σ(*F*^2^)] = 0.043	Hydrogen site location: inferred from neighbouring sites
*wR*(*F*^2^) = 0.100	H-atom parameters constrained
*S* = 1.01	*w* = 1/[σ^2^(*F*_o_^2^) + (0.0416*P*)^2^] where *P* = (*F*_o_^2^ + 2*F*_c_^2^)/3
3358 reflections	(Δ/σ)_max_ = 0.001
289 parameters	Δρ_max_ = 0.98 e Å^−^^3^
6 restraints	Δρ_min_ = −1.14 e Å^−^^3^

### Special details

**Table:**

Geometry. All e.s.d.'s (except the e.s.d. in the dihedral angle between two l.s. planes) are estimated using the full covariance matrix. The cell e.s.d.'s are taken into account individually in the estimation of e.s.d.'s in distances, angles and torsion angles; correlations between e.s.d.'s in cell parameters are only used when they are defined by crystal symmetry. An approximate (isotropic) treatment of cell e.s.d.'s is used for estimating e.s.d.'s involving l.s. planes.
Refinement. Refinement of *F*^2^ against ALL reflections. The weighted *R*-factor *wR* and goodness of fit *S* are based on *F*^2^, conventional *R*-factors *R* are based on *F*, with *F* set to zero for negative *F*^2^. The threshold expression of *F*^2^ > σ(*F*^2^) is used only for calculating *R*-factors(gt) *etc*. and is not relevant to the choice of reflections for refinement. *R*-factors based on *F*^2^ are statistically about twice as large as those based on *F*, and *R*- factors based on ALL data will be even larger.

### Fractional atomic coordinates and isotropic or equivalent isotropic displacement parameters (Å^2^)

**Table:**

	*x*	*y*	*z*	*U*_iso_*/*U*_eq_	
Mo1	−0.16871 (9)	1.12585 (6)	0.46162 (6)	0.00650 (17)	
Mo2	0.11157 (9)	1.09175 (6)	0.19220 (6)	0.00741 (17)	
Mo3	0.26045 (9)	1.15843 (6)	0.40116 (6)	0.00665 (17)	
Mo4	−0.28939 (9)	1.02948 (6)	0.25245 (6)	0.00758 (18)	
O1	−0.3420 (7)	0.8971 (5)	0.2506 (5)	0.0133 (12)	
O2	−0.3693 (7)	1.1971 (4)	0.5145 (5)	0.0105 (12)	
O3	0.1859 (7)	1.2201 (4)	0.2416 (5)	0.0085 (11)	
O4	0.0616 (7)	1.2015 (5)	0.0567 (5)	0.0128 (12)	
O5	0.3085 (7)	1.0059 (5)	0.1528 (5)	0.0137 (12)	
O6	−0.2454 (7)	0.9638 (4)	0.4425 (5)	0.0104 (12)	
O7	0.2797 (7)	1.2922 (5)	0.4247 (5)	0.0115 (12)	
O8	−0.0349 (7)	0.9717 (4)	0.2159 (5)	0.0083 (11)	
O9	−0.1648 (7)	1.1673 (4)	0.2906 (5)	0.0096 (11)	
O10	0.1167 (7)	1.0169 (4)	0.3927 (5)	0.0094 (11)	
O11	−0.0233 (7)	1.2188 (4)	0.4667 (5)	0.0100 (11)	
O12	−0.3228 (7)	1.1403 (5)	0.1127 (5)	0.0139 (12)	
O13	0.4808 (7)	1.0993 (5)	0.3414 (5)	0.0112 (12)	
N1	0.2675 (9)	0.4044 (6)	0.0410 (6)	0.0101 (14)	
H1A	0.2439	0.3421	0.1048	0.012*	
N2	0.2821 (9)	0.5971 (6)	−0.0789 (6)	0.0163 (16)	
H2A	0.2683	0.6778	−0.1040	0.020*	
N3	0.0301 (9)	0.7334 (6)	0.2313 (6)	0.0129 (15)	
H3A	0.0292	0.8091	0.2287	0.016*	
C1	0.3678 (11)	0.5190 (7)	−0.1506 (7)	0.0111 (17)	
C2	0.4479 (11)	0.5449 (7)	−0.2742 (7)	0.0164 (19)	
H2B	0.4529	0.6267	−0.3251	0.020*	
C3	0.5193 (11)	0.4427 (8)	−0.3171 (8)	0.0184 (19)	
H3B	0.5757	0.4556	−0.3986	0.022*	
C4	0.5087 (10)	0.3195 (7)	−0.2404 (7)	0.0134 (17)	
H4A	0.5561	0.2535	−0.2738	0.016*	
C5	0.4315 (11)	0.2924 (7)	−0.1185 (7)	0.0161 (18)	
H5A	0.4289	0.2101	−0.0682	0.019*	
C6	0.3569 (10)	0.3953 (7)	−0.0735 (7)	0.0121 (17)	
C7	0.2234 (10)	0.5267 (7)	0.0365 (7)	0.0107 (17)	
C8	0.1258 (10)	0.5724 (6)	0.1383 (7)	0.0110 (17)	
C9	0.1228 (10)	0.6933 (7)	0.1385 (7)	0.0075 (16)	
H9A	0.1863	0.7462	0.0729	0.009*	
C10	−0.0629 (12)	0.6621 (7)	0.3295 (7)	0.0176 (19)	
H10A	−0.1266	0.6943	0.3931	0.021*	
C11	−0.0631 (11)	0.5392 (7)	0.3352 (7)	0.0152 (18)	
H11A	−0.1241	0.4876	0.4038	0.018*	
C12	0.0270 (12)	0.4951 (7)	0.2394 (8)	0.0182 (19)	
H12A	0.0234	0.4144	0.2406	0.022*	

### Atomic displacement parameters (Å^2^)

**Table:**

	*U*^11^	*U*^22^	*U*^33^	*U*^12^	*U*^13^	*U*^23^
Mo1	0.0066 (4)	0.0062 (3)	0.0058 (3)	−0.0016 (3)	−0.0010 (3)	−0.0003 (2)
Mo2	0.0072 (4)	0.0076 (3)	0.0064 (3)	−0.0024 (3)	−0.0002 (3)	−0.0007 (2)
Mo3	0.0067 (4)	0.0060 (3)	0.0063 (3)	−0.0027 (3)	−0.0014 (3)	0.0005 (2)
Mo4	0.0078 (4)	0.0079 (3)	0.0066 (4)	−0.0027 (3)	−0.0011 (3)	−0.0007 (2)
O1	0.018 (3)	0.012 (3)	0.010 (3)	−0.007 (2)	−0.004 (2)	−0.001 (2)
O2	0.007 (3)	0.011 (3)	0.012 (3)	0.001 (2)	−0.001 (2)	−0.002 (2)
O3	0.009 (3)	0.007 (3)	0.009 (3)	−0.001 (2)	−0.003 (2)	0.000 (2)
O4	0.013 (3)	0.013 (3)	0.012 (3)	−0.004 (2)	−0.004 (2)	−0.001 (2)
O5	0.013 (3)	0.011 (3)	0.015 (3)	0.003 (2)	−0.003 (3)	−0.006 (2)
O6	0.010 (3)	0.013 (3)	0.009 (3)	−0.003 (2)	−0.003 (2)	−0.001 (2)
O7	0.011 (3)	0.014 (3)	0.009 (3)	−0.001 (2)	−0.001 (2)	−0.005 (2)
O8	0.007 (3)	0.007 (2)	0.009 (3)	−0.004 (2)	0.002 (2)	0.000 (2)
O9	0.013 (3)	0.007 (3)	0.006 (3)	−0.002 (2)	−0.001 (2)	0.002 (2)
O10	0.012 (3)	0.007 (3)	0.009 (3)	0.000 (2)	−0.006 (2)	0.000 (2)
O11	0.011 (3)	0.007 (3)	0.012 (3)	0.000 (2)	−0.006 (2)	−0.002 (2)
O12	0.015 (3)	0.015 (3)	0.011 (3)	−0.004 (2)	−0.002 (2)	−0.002 (2)
O13	0.006 (3)	0.017 (3)	0.010 (3)	−0.004 (2)	−0.003 (2)	−0.001 (2)
N1	0.014 (4)	0.010 (3)	0.004 (3)	−0.005 (3)	0.004 (3)	−0.002 (2)
N2	0.023 (4)	0.003 (3)	0.019 (4)	0.005 (3)	−0.008 (3)	0.001 (3)
N3	0.012 (4)	0.006 (3)	0.020 (4)	0.003 (3)	−0.007 (3)	−0.004 (3)
C1	0.014 (4)	0.008 (4)	0.010 (4)	−0.004 (3)	−0.002 (3)	0.000 (3)
C2	0.021 (5)	0.015 (4)	0.008 (4)	−0.008 (4)	0.003 (4)	0.004 (3)
C3	0.012 (5)	0.023 (5)	0.023 (5)	−0.001 (4)	−0.008 (4)	−0.009 (4)
C4	0.006 (4)	0.016 (4)	0.018 (5)	−0.001 (3)	−0.001 (4)	−0.007 (3)
C5	0.024 (5)	0.015 (4)	0.013 (4)	−0.010 (4)	−0.003 (4)	−0.004 (3)
C6	0.009 (4)	0.016 (4)	0.016 (4)	−0.005 (3)	−0.006 (4)	−0.007 (3)
C7	0.011 (4)	0.007 (4)	0.013 (4)	−0.002 (3)	0.000 (3)	−0.003 (3)
C8	0.010 (4)	0.003 (3)	0.015 (4)	0.003 (3)	−0.002 (3)	0.002 (3)
C9	0.003 (4)	0.011 (4)	0.006 (4)	−0.001 (3)	−0.001 (3)	0.001 (3)
C10	0.024 (5)	0.018 (4)	0.009 (4)	−0.001 (4)	0.000 (4)	−0.007 (3)
C11	0.019 (5)	0.004 (4)	0.016 (4)	0.003 (3)	−0.002 (4)	0.002 (3)
C12	0.026 (5)	0.013 (4)	0.018 (5)	−0.008 (4)	−0.009 (4)	−0.001 (3)

### Geometric parameters (Å, °)

**Table:**

Mo1—O2	1.690 (5)	N1—H1A	0.8600
Mo1—O11	1.776 (5)	N2—C7	1.353 (10)
Mo1—O9	1.875 (5)	N2—C1	1.386 (10)
Mo1—O10^i^	1.956 (5)	N2—H2A	0.8600
Mo1—O6	2.189 (5)	N3—C9	1.317 (10)
Mo1—O10	2.416 (5)	N3—C10	1.339 (11)
Mo2—O5	1.693 (5)	N3—H3A	0.8600
Mo2—O4	1.709 (5)	C1—C2	1.395 (11)
Mo2—O8	1.927 (5)	C1—C6	1.410 (10)
Mo2—O3	2.004 (5)	C2—C3	1.378 (11)
Mo2—O10	2.200 (5)	C2—H2B	0.9300
Mo2—O9	2.345 (5)	C3—C4	1.404 (11)
Mo3—O7	1.699 (5)	C3—H3B	0.9300
Mo3—O13	1.790 (5)	C4—C5	1.371 (11)
Mo3—O6^i^	1.880 (5)	C4—H4A	0.9300
Mo3—O3	1.922 (5)	C5—C6	1.402 (11)
Mo3—O11	2.229 (6)	C5—H5A	0.9300
Mo3—O10	2.242 (5)	C7—C8	1.445 (11)
Mo4—O1	1.682 (5)	C8—C9	1.385 (10)
Mo4—O12	1.719 (5)	C8—C12	1.413 (11)
Mo4—O8	1.965 (5)	C9—H9A	0.9300
Mo4—O13^ii^	2.012 (5)	C10—C11	1.393 (11)
Mo4—O6	2.160 (5)	C10—H10A	0.9300
Mo4—O9	2.266 (5)	C11—C12	1.363 (11)
N1—C7	1.350 (9)	C11—H11A	0.9300
N1—C6	1.384 (10)	C12—H12A	0.9300
			
O2—Mo1—O11	104.1 (2)	Mo3^i^—O6—Mo4	149.5 (3)
O2—Mo1—O9	104.5 (2)	Mo3^i^—O6—Mo1	107.8 (2)
O11—Mo1—O9	101.6 (2)	Mo4—O6—Mo1	102.4 (2)
O2—Mo1—O10^i^	101.7 (2)	Mo2—O8—Mo4	116.1 (2)
O11—Mo1—O10^i^	98.2 (2)	Mo1—O9—Mo4	109.5 (2)
O9—Mo1—O10^i^	142.0 (2)	Mo1—O9—Mo2	111.1 (2)
O2—Mo1—O6	98.6 (2)	Mo4—O9—Mo2	91.48 (18)
O11—Mo1—O6	156.9 (2)	Mo1^i^—O10—Mo2	149.5 (3)
O9—Mo1—O6	76.6 (2)	Mo1^i^—O10—Mo3	103.1 (2)
O10^i^—Mo1—O6	72.61 (19)	Mo2—O10—Mo3	96.11 (19)
O2—Mo1—O10	177.8 (2)	Mo1^i^—O10—Mo1	103.8 (2)
O11—Mo1—O10	76.9 (2)	Mo2—O10—Mo1	98.11 (18)
O9—Mo1—O10	77.1 (2)	Mo3—O10—Mo1	93.98 (18)
O10^i^—Mo1—O10	76.2 (2)	Mo1—O11—Mo3	116.2 (2)
O6—Mo1—O10	80.29 (19)	Mo3—O13—Mo4^iii^	170.5 (3)
O5—Mo2—O4	105.2 (3)	C7—N1—C6	109.3 (6)
O5—Mo2—O8	98.6 (2)	C7—N1—H1A	125.4
O4—Mo2—O8	102.0 (2)	C6—N1—H1A	125.4
O5—Mo2—O3	101.2 (2)	C7—N2—C1	109.5 (6)
O4—Mo2—O3	90.8 (2)	C7—N2—H2A	125.2
O8—Mo2—O3	152.7 (2)	C1—N2—H2A	125.2
O5—Mo2—O10	94.9 (2)	C9—N3—C10	123.3 (7)
O4—Mo2—O10	156.3 (2)	C9—N3—H3A	118.3
O8—Mo2—O10	87.0 (2)	C10—N3—H3A	118.3
O3—Mo2—O10	72.70 (19)	N2—C1—C2	131.8 (7)
O5—Mo2—O9	165.8 (2)	N2—C1—C6	106.1 (7)
O4—Mo2—O9	88.3 (2)	C2—C1—C6	122.1 (7)
O8—Mo2—O9	73.7 (2)	C3—C2—C1	116.4 (7)
O3—Mo2—O9	82.7 (2)	C3—C2—H2B	121.8
O10—Mo2—O9	73.09 (18)	C1—C2—H2B	121.8
O7—Mo3—O13	103.0 (2)	C2—C3—C4	121.5 (8)
O7—Mo3—O6^i^	106.4 (2)	C2—C3—H3B	119.3
O13—Mo3—O6^i^	97.5 (2)	C4—C3—H3B	119.3
O7—Mo3—O3	102.6 (2)	C5—C4—C3	122.8 (8)
O13—Mo3—O3	93.5 (2)	C5—C4—H4A	118.6
O6^i^—Mo3—O3	145.6 (2)	C3—C4—H4A	118.6
O7—Mo3—O11	82.9 (2)	C4—C5—C6	116.5 (7)
O13—Mo3—O11	173.7 (2)	C4—C5—H5A	121.7
O6^i^—Mo3—O11	82.6 (2)	C6—C5—H5A	121.7
O3—Mo3—O11	83.0 (2)	N1—C6—C5	132.6 (7)
O7—Mo3—O10	155.7 (2)	N1—C6—C1	106.7 (7)
O13—Mo3—O10	101.2 (2)	C5—C6—C1	120.6 (8)
O6^i^—Mo3—O10	72.7 (2)	N1—C7—N2	108.4 (7)
O3—Mo3—O10	73.22 (19)	N1—C7—C8	124.8 (7)
O11—Mo3—O10	72.83 (19)	N2—C7—C8	126.7 (7)
O1—Mo4—O12	106.8 (2)	C9—C8—C12	118.2 (7)
O1—Mo4—O8	94.3 (2)	C9—C8—C7	121.4 (7)
O12—Mo4—O8	99.6 (2)	C12—C8—C7	120.4 (7)
O1—Mo4—O13^ii^	98.9 (3)	N3—C9—C8	120.2 (7)
O12—Mo4—O13^ii^	92.9 (2)	N3—C9—H9A	119.9
O8—Mo4—O13^ii^	158.5 (2)	C8—C9—H9A	119.9
O1—Mo4—O6	97.2 (2)	N3—C10—C11	119.1 (8)
O12—Mo4—O6	155.4 (2)	N3—C10—H10A	120.4
O8—Mo4—O6	84.1 (2)	C11—C10—H10A	120.4
O13^ii^—Mo4—O6	77.4 (2)	C12—C11—C10	119.5 (8)
O1—Mo4—O9	163.5 (2)	C12—C11—H11A	120.2
O12—Mo4—O9	87.6 (2)	C10—C11—H11A	120.2
O8—Mo4—O9	74.95 (19)	C11—C12—C8	119.7 (7)
O13^ii^—Mo4—O9	88.2 (2)	C11—C12—H12A	120.2
O6—Mo4—O9	69.69 (18)	C8—C12—H12A	120.2
Mo3—O3—Mo2	114.6 (2)		

Symmetry codes: (i) −*x*, −*y*+2, −*z*+1; (ii) *x*−1, *y*, *z*; (iii) *x*+1, *y*, *z*.

### Hydrogen-bond geometry (Å, °)

**Table:**

*D*—H···*A*	*D*—H	H···*A*	*D*···*A*	*D*—H···*A*
N1—H1A···O3^iv^	0.86	1.78	2.639 (8)	176
N3—H3A···O8	0.86	1.78	2.614 (8)	164

Symmetry codes: (iv) *x*, *y*−1, *z*.
